# Draft Genome Sequence and Assembly of *Photorhabdus heterorhabditis* Strain VMG, a Bacterial Symbiont Associated with the Entomopathogenic Nematode *Heterorhabditis zealandica*

**DOI:** 10.1128/genomeA.01279-15

**Published:** 2015-10-29

**Authors:** Stephanie Naidoo, Boipelo Mothupi, Jonathan Featherston, Phelelani T. Mpangase, Vincent M. Gray

**Affiliations:** a School of Molecular and Cell Biology, University of the Witwatersrand, Johannesburg, South Africa; b Biotechnology Platform, Agricultural Research Council, Onderstepoort, South Africa; c Sydney Brenner Institute for Molecular Bioscience (SBIMB), Wits 21st Century Institutes Wits Bioinformatics, Johannesburg, South Africa

## Abstract

Here, we report the draft genome sequence of *Photorhabdus heterorhabditis* strain VMG, a symbiont of the entomopathogenic nematode *Heterorhabditis zealandica* in South Africa. The draft genome sequence is 4,878,919 bp long and contains 4,023 protein-coding genes. The genome assembly contains 262 contigs with a G+C content of 42.22%.

## GENOME ANNOUNCEMENT

The genus *Photorhabdus* consists of bioluminescent Gram-negative bacteria, which are lethal insect pathogens to the larval stage of numerous insect orders such as *Lepidoptera*, *Coleopteran*, *Hymenoptera*, and *Dictyoptera* (1, 2). A striking feature observed in members of *Photorhabdus* is their ability to forge symbiotic associations with entomopathogenic nematodes of the family *Heterorhabditidae*.

The lifestyle of *Photorhabdus* largely depends on the free-living infective stage of their nematode hosts, referred to as infective juveniles (IJs), which serve as vectors for the transmission of *Photorhabdus* among susceptible insects (3). The IJ nematodes actively search for susceptible, soil-dwelling insects to parasitize. Upon finding such a host, the IJs gain entry into the hemocoel of the insect where they release their enteric symbionts into the hemolymph. The bacteria rapidly proliferate and are able to elude the immune response of the insect (4, 5). Through the release of highly virulent bacterial toxins (6, 7), the insect succumbs to death within 48 h. During the stationary phase of its growth cycle, the bacteria also secrete extracellular enzymes that facilitate the breakdown of insect tissue into a nutrient soup, which in turn sustains the bacteria and nematode development (8, 9).

The nematode-bacterium partnership is capable of invading and killing a broad range of insect pests, thus having great agricultural importance. Here, we present the draft genome sequence for *P. heterorhabditis* strain VMG, which was isolated from *Heterorhabditis zealandica* nematodes found in South Africa.

The entomopathogen, *P. heterorhabditis* strain VMG was isolated, according to the method described by Kaya and Stock (10). Genomic DNA was extracted using the ZR fungal/bacterial DNA miniprep kit (Zymo Research). Paired-end libraries were constructed using the Nextera DNA sample preparation kit (Illumina). Paired-end (2 × 300 bp) sequencing was performed on a MiSeq instrument (Illumina) using a MiSeq reagent kit version 3, which resulted in 5,381,836 reads. Adapter and quality trimming was performed using Trimmomatic version 0.32 (11). The pre-processed reads were assembled *de novo* using SPAdes version 3.5.0 (12) and the assembly was assessed using QUAST version 3.1 (13). Trimmed reads also were mapped to the final assembly to calculate the read depth using CLC Genomics Workbench version 8.0.3. The assembled genome was annotated using the NCBI Prokaryotic Genome Automatic Annotation Pipeline (PGAAP) (14).

The final assembly consisted of 262 contigs (234 of which were above 500 bp), with a *N*_50_ size of 59,494 bp and a G+C content of 42.22%. The genome size of *P. heterorhabditi*s strain VMG was found to be 4,878,919 bp at 115× coverage. Annotation revealed 4,343 predicted genes and 4,023 protein-coding sequences (CDS), including 229 pseudogenes and 72 tRNAs being identified.

The genome sequence of *P. heterorhabditis* may pave the way for more intensive studies on the virulence mechanisms and antimicrobial properties of this entomopathogen. Furthermore, it also promises to be a genomic resource for discovering novel insecticidal agents.

### Nucleotide sequence accession numbers. 

This whole-genome shotgun project has been deposited at DDBJ/EMBL/GenBank under the accession no. LJCS00000000. The version described in this paper is version LJCS01000000.
